# Appropriate dose reduction using photon-counting detector CT for temporal bone imaging: phantom and clinical studies with helical acquisition

**DOI:** 10.1007/s11604-026-02002-9

**Published:** 2026-05-20

**Authors:** Yuka Takahashi, Fumiyo Higaki, Yuko Nakamura, Toru Higaki, Akiko Sugaya, Toshiharu Mitsuhashi, Takahiro Kitayama, Yusuke Morimitsu, Tomohiro Inoue, Noriaki Akagi, Yusuke Matsui, Toshihiro Iguchi, Kazuo Awai, Takao Hiraki

**Affiliations:** 1 Department of Radiology, Okayama University Graduate School of Medicine, Dentistry and Pharmaceutical Sciences, Okayama, Japan; 2 Department of Radiology, Medical Development Field, Okayama University, 2-5-1 Shikata-Cho, Kitaku, Okayama, 700-8558 Japan; 3 Department of Diagnostic Radiology, Hiroshima University, Hiroshima, Japan; 4 Graduate School of Advanced Science and Engineering, Hiroshima University, Hiroshima, Japan; 5 Department of Otolaryngology-Head and Neck Surgery, Faculty of Medicine, Dentistry, and Pharmaceutical Sciences, Okayama University, Okayama, Japan; 6 Center for Innovative Clinical Medicine, Okayama University, Okayama, Japan; 7 Department of Radiological Technology, Okayama University Hospital, Okayama, Japan; 8 Department of Radiology, Faculty of Medicine, Dentistry, and Pharmaceutical Sciences, Okayama University, Okayama, Japan; 9 Department of Radiological Technology, Faculty of Health Sciences, Okayama University, Okayama, Japan

**Keywords:** PCD-CT, EID-CT, Dose reduction, Temporal bone CT, Incudomalleolar joint, Stapes

## Abstract

**Purpose:**

To determine the extent of possible dose reduction with photon-counting detector computed tomography (PCD-CT) while maintaining image quality equivalent to that of energy-integrating detector CT (EID-CT) images at standard dose in the temporal bone.

**Materials and methods:**

PCD-CT and EID-CT imaging quality were compared by visual evaluation of clinical temporal bone images and visual scores with Welch’s t-test at standard dose. A head phantom was used to evaluate imaging quality under dose reduction. The detectability index (d’) of the PCD-CT images at various dose levels and the EID-CT images at standard dose was evaluated. Dose reduction limit with PCD-CT used in the subsequent clinical evaluation was determined as the lowest dose with image quality equal to or better than EID-CT. The clinical equivalence of PCD-CT image quality at the determined reduced dose to that with EID-CT at standard dose was evaluated using visual scores. Equivalence was determined if the 95% confidence intervals of differences did not exceed the equivalence margin of ±1.

**Results:**

At standard doses, PCD-CT images demonstrated significantly higher visual scores than EID-CT images (3.73 vs. 2.56 for incudomalleolar joint, 3.75 vs. 2.63 for stapes, 3.54 vs. 2.52 for cochlea, and 3.58 vs. 2.46 for facial nerve canal; all P 0.001). In the phantom study, the d’ value was 0.15 with EID-CT at standard dose and was 0.12 and 0.17 with PCD-CT at 25% and 50% of the standard dose, respectively. Clinically, the mean visual scores of PCD-CT images at 50% of the standard dose were equivalent to EID-CT images at standard dose in all regions (3.58 vs. 3.12 for incudomalleolar joint, 3.46 vs. 3.19 for stapes, 3.50 vs. 3.08 for cochlea, 3.58 vs. 3.27 for facial nerve canal).

**Conclusion:**

PCD-CT may preserve image quality even at 50% of the standard dose in the temporal bone.

## Introduction

Energy-integrating detector computed tomography (EID-CT) is a conventional CT technique that has undergone various advancements to produce higher resolution images. Among these, ultra-high-resolution (UHR) multidetector CT can visualize the microstructure of bone trabeculae with higher resolution [1]. In temporal bone imaging, previous studies have demonstrated that UHR-CT using energy-integrating detector systems enables improved visualization of fine osseous structures, including the ossicular chain and the stapes footplate [2, 3]. Recently, a novel CT system equipped with a different type of detector, photon-counting detector CT (PCD-CT), was developed. The main advantages of PCD-CT over conventional EID-CT include dose reduction, improved spatial resolution, and reduced artifacts [4–6]. For example, Baffour et al. reported that PCD-CT provided superior visualization of bony structures in the shoulder and pelvis, even at a 31–47% lower radiation dose compared to EID-CT [7]. Booij et al. showed that PCD-CT improved the delineation of bone structures and microfractures in the hand and wrist [8]. The usefulness of PCD-CT has also been reported in temporal bone regions; it has been shown to provide better temporal bone anatomy, including the tympanic membrane, stapedial head, incudostapedial joint, cortical margin of the tympanic segment of the facial nerve, and the cochlear modiolus [9]. Benson et al. also demonstrated that temporal bone images with PCD-CT at a 31% dose reduction showed better spatial resolution and delineation of key structures than conventional EID-CT [10]. However, the specific dose reduction limits while maintaining image quality were not determined in their study. Thus, this study aimed to determine specific dose reduction values with PCD-CT while ensuring image quality equivalent to that of EID-CTs.

## Materials and methods

All procedures involving human participants were approved by the Okayama University Ethics Committee (approval numbers: K2402-016) and were conducted in accordance with the Japanese Ethical Guidelines for Medical and Biological Research Involving Human Subjects and the 1964 Declaration of Helsinki and its later amendments.

This study comprised three components. The first component was a retrospective clinical evaluation of pre-existing temporal bone images: the quality of PCD-CT (NAEOTOM Alpha; Siemens Healthineer, Erlangen, Germany) images at standard dose was compared with that of EID-CT (Aquilion Precision; Canon Medical Systems, Otawara, Japan) images at standard dose. The second component was a phantom experiment, where we evaluated how much dose reduction was possible with PCD-CT while maintaining an image quality equivalent to that of the EID-CT images at standard dose. The third component was a clinical evaluation of PCD-CT images obtained at the determined reduced dose, which were compared with pre-existing EID-CT images at standard dose in terms of imaging quality.

### Determining the required sample size for the clinical evaluation

An unpublished pilot study, where the incudomallear joint, stapes, cochlea, and facial nerve canal were evaluated in ten patients, was conducted to determine the required sample size for the clinical evaluation. Data from an unpublished pilot study, were used to estimate the sample size required for the first study component; the data included the mean (± standard deviation [SD]) of visual scores of 2.4 (± 0.70) for EID-CT and 3.1 (± 0.74) for PCD-CT. Based on these values, with α = 0.05 and power = 0.80, the required sample size was calculated to be at least 18 per group. In order to enhance the reliability of the results, the final size was set at 24 patients per group.

For the third study component, it was assumed that the score for the incudomalleolar joint after dose reduction with PCD-CT would be 2.4 (± 0.70), equivalent to EID-CT. The equivalence margin was set at 1, with α = 0.05 and β = 0.20. The required sample size was calculated to be at least nine per group. In order to enhance the reliability of the results, the final size was set at 13 patients per group.

### Retrospective clinical evaluation of pre-existing temporal bone images: PCD-CT vs. EID-CT

**Fig. 1:**
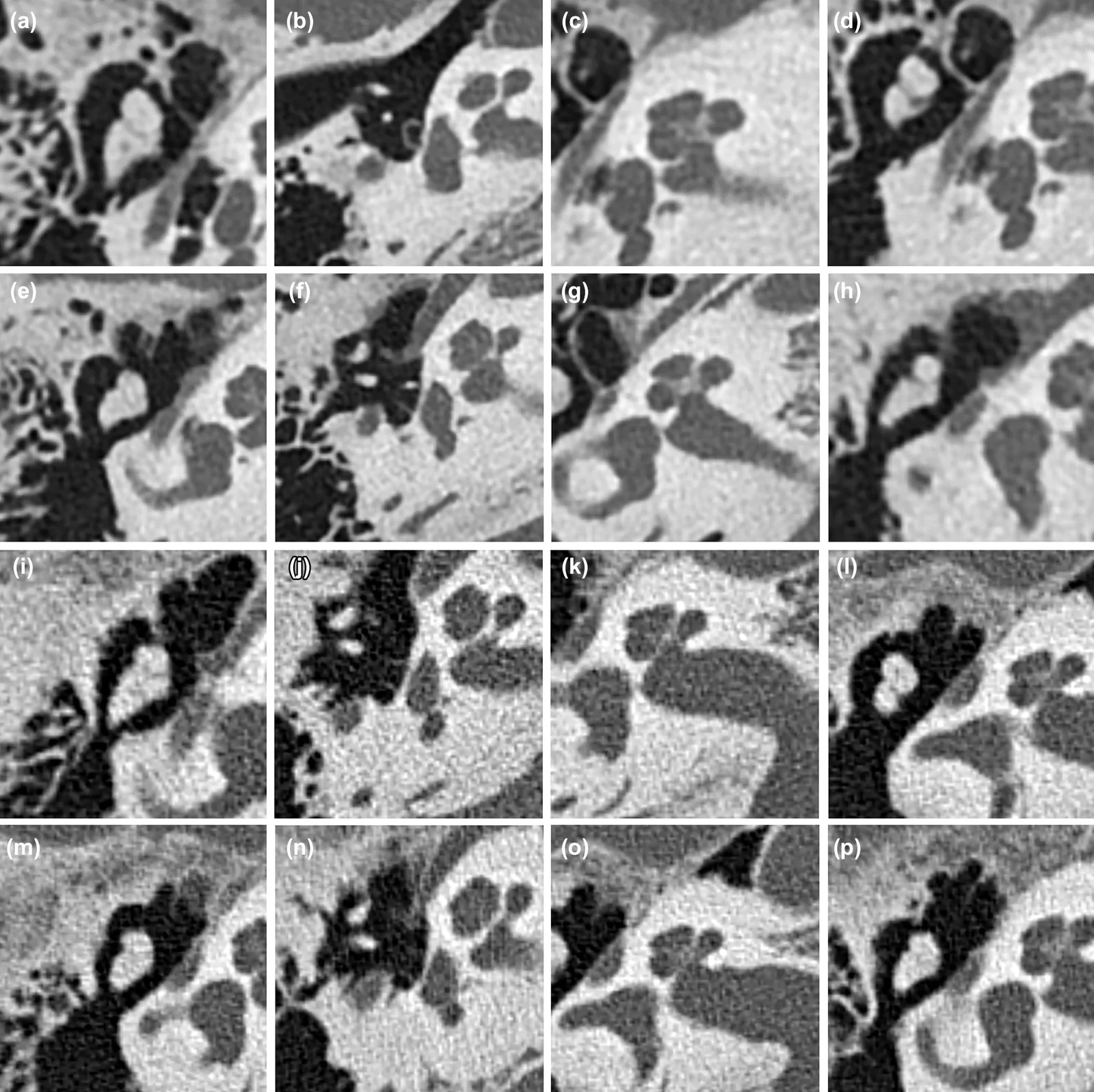
Examples of scores 4 (**a**–**d**), 3 (**e**–**h**), 2 (**i**–**l**) and 1 (**m**–**p**) used for visual assessment. Image quality progressively improves from score 1 to score 4 for all evaluated structures. The incudomalleolar joint, stapes (anterior and posterior crura), cochlea (including the modiolus), and facial nerve canal are increasingly well delineated with higher scores. Score 4 demonstrates clear depiction, whereas score 1 shows marked blurring and noise-related obscuration

Consecutive 24 adult patients who underwent temporal bone CT with both PCD-CT and EID-CT at standard dose between December 2022 and June 2023 were included. The interval and sequence of the CT scans was not predetermined, but were based on clinical indications. Patients with bilateral abnormalities of the inner or middle ear were excluded. All 24 patients provided informed consent via an opt-out process. The CT parameters are shown in Table 1. The quality of the two images was compared using visual scores, which were evaluated in four regions: the incudomallear joint, stapes, cochlea, and facial nerve canal, by two blinded and independent radiologists with 19 (F.H.) and 10 (T.K.) years of experience in diagnostic radiology. When both sides were normal, the left side was evaluated, whereas in cases of unilateral abnormality, the normal side was assessed. The scores were divided into five categories: 4, excellent; 3, good; 2, normal; 1, poor; and 0, non-evaluative. Examples of scores 1–4 are shown in Fig. 1.

**Table 1:** CT parameters of the clinical cases

	EID-CT at standard dose	PCD-CT at standard dose	PCD-CT at the reduced dose
Collimation (mm)	160*0.25-SHRmode	120*0.2-UHRmode	
Tube voltage (kV)	120	120	
Tube current (mA)	170–200		
Quality ref.mAs		252	126
Rotation time (s)	1.0	0.5	
Pitch	0.569–0.679	0.55	
CTDIvol (mGy)	39.4–46.3	40.9–53.9	19.4–23.9
Slice thickness (mm)	0.25	0.2	0.2
Slice interval (mm)	0.25	0.1	0.2
Bone kernel	FC81(AIDR 3D eMild)	Hr76(QIR 1)	
Field of view (mm)	100		
Window width/level (HU)	4000/600		

CT, computed tomography; PCD-CT, photon-counting detector computed tomography; EID-CT, energy-integrating detector computed tomography; CTDIvol, Computed Tomography Dose Index volume; HU, Hounsfield Unit; SHR, Super High Resolution; UHR, Ultra High Resolution

### Phantom experiment to determine dose reduction limit with PCD-CT

**Fig. 2:**
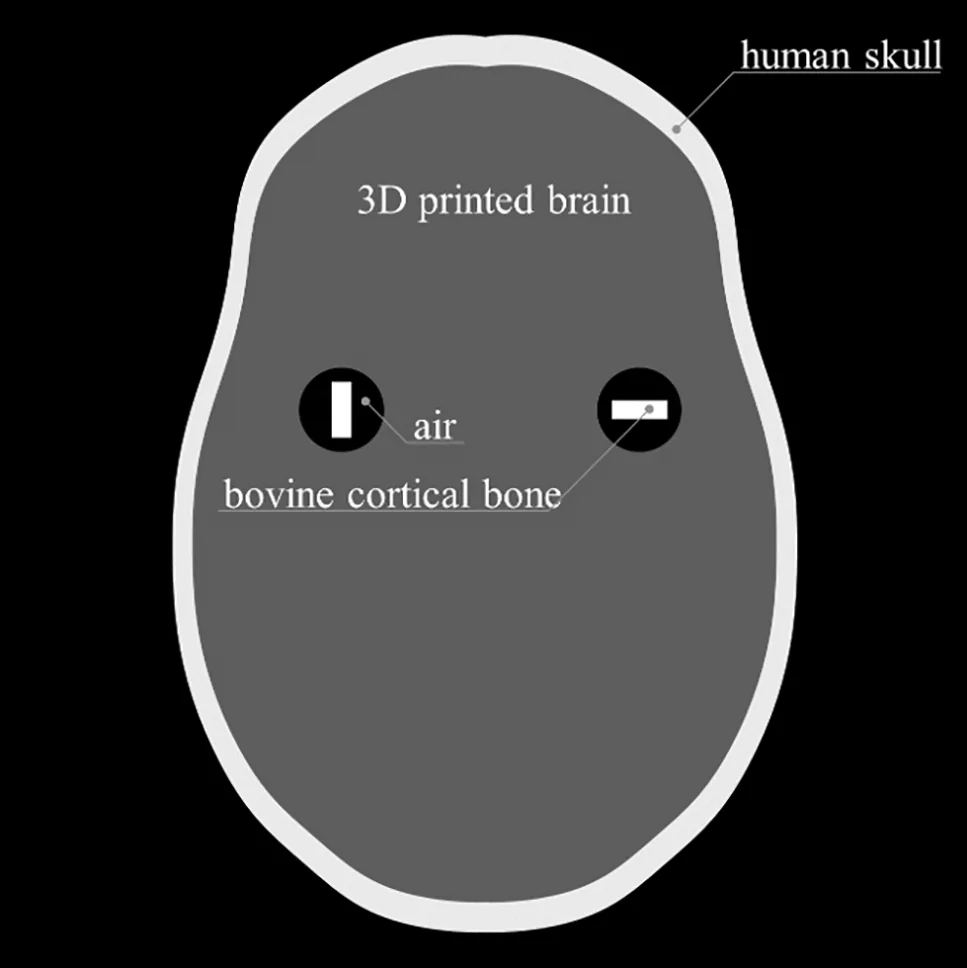
Design of our head phantom created using a 3D printer. The phantom is composed of simulated brain parenchyma within a human skull. A cavity is created within the simulated brain parenchyma to accommodate the bovine cortical bone, which simulates the auditory ossicles

A homemade head phantom (Fig. 2) was used for the phantom experiment. A human skull was obtained from the Department of Anatomy, where it had been stored for educational and research purposes. Simulated brain parenchyma was created using a 3D printer and then inserted inside the human skull. Cavities were created within the simulated brain parenchyma to accommodate the bovine cortical bones, which simulated the auditory ossicles.

The phantom was scanned by PCD-CT with the imaging protocols shown in Table 2. PCD-CT scanned with the same radiation exposure as the clinical conventional protocol was defined as “PCD at standard dose.” The reduced dose protocols included “PCD at 50% dose” (half the standard radiation dose) and “PCD at 25% dose” (one-fourth of the radiation dose), which were achieved by adjusting the tube current. The phantom was also scanned by EID-CT with the standard dose as the control. Image quality was evaluated using the detectability index d’, which was expressed as a composite function of metrics related to spatial resolution and image noise, thereby quantifying the detectability of a specific diagnostic task rather than evaluating image noise or resolution separately [11]. Spatial resolution was calculated as the mean of the horizontal and vertical values obtained from the bovine cortical bone simulating the auditory ossicles. The noise metric was measured from uniform regions of the simulated brain parenchyma. The target spatial frequency was 1.0 lp/mm. The d’ values were calculated using a custom program developed for ImageJ. The dose reduction limit with PCD-CT was determined as the lowest dose with image quality equal to or better than EID-CT and then used in the subsequent clinical evaluation.

**Table 2:** CT parameters in the phantom experiment

Scanner at dose		Detector (mm × raw)	Tube voltage (kV)	Tube current (mA)	Rotation time (ms)	CTDI vol (mGy)	Focus	Reconstruction	Kernel	Matrix
EID-CT at standard dose		0.25 × 160	120	170	1000	39.4	0.5 × 0.4	Hybrid IR	FC81(AIDR 3D eMild)	1024
PCD-CT at standard dose		0.2 × 120	120	255	500	39.8	0.6 × 0.7	Hybrid IR	Hr76(QIR 1)	1024
PCD-CT at 50% dose		0.2 × 120	120	128	500	20.0	0.6 × 0.7	Hybrid IR	Hr76(QIR 1)	1024
PCD-CT at 25% dose		0.2 × 120	120	64	500	10.0	0.6 × 0.7	Hybrid IR	Hr76(QIR 1)	1024

CT, computed tomography; PCD-CT, photon-counting detector computed tomography; EID-CT, energy-integrating detector computed tomography; IR, Iterative Reconstruction; AIDR, Adaptive Iterative Dose Reduction

### Clinical evaluation of PCD-CT images at the determined reduced dose

PCD-CT images at the determined reduced dose were validated in clinical cases. Thus, the equivalence of PCD-CT images at the reduced dose to EID-CT images at standard dose was evaluated. Thirteen patients were prospectively enrolled from April 2024 to May 2025 and underwent scans by PCD-CT at the reduced dose with parameters shown in Table 1. Written informed consent was obtained from all patients who underwent low-dose PCD-CT. EID-CT images at standard dose acquired from 13 consecutive patients between March and June 2023 were retrospectively collected as control. The quality of the two images was compared with the same method using the visual scores mentioned above.

### Statistical analyses

In the first study component, the mean of the scores given by the two readers was calculated for each participant, and the mean values were compared between the PCD-CT and EID-CT groups using Welch’s t-test. Statistical significance was set at *P* < 0.05. The mean differences between the groups and their 95% confidence intervals (CI) were also calculated.

In the third study component, the mean differences and their 95% CI of visual scores between PCD-CT images at the reduced dose and EID-CT images at the standard dose were calculated. If the 95% CI for the difference did not exceed the equivalence margin of ± 1, equivalence was concluded.

In the first and third study components, inter-reader agreement was assessed using weighted kappa (κ) coefficients. The weights were defined as 1 − {(i − j)/(k − 1)}^2^, where i and j index the rows and columns of the rating matrix, and k is the total number of possible rating categories. According to the classification proposed by Landis and Koch [12], the κ values were interpreted as follows: < 0.00, Poor; 0.00–0.20, Slight; 0.21–0.40, Fair; 0.41–0.60, Moderate; 0.61–0.80, Substantial; and 0.81–1.00, Almost perfect.

Statistical analysis was performed using Stata/MP 19.5 (StataCorp LLC, College Station, TX, USA).

## Results

### Retrospective clinical evaluation of pre-existing temporal bone images: PCD-CT vs. EID-CT

**Fig. 3:**
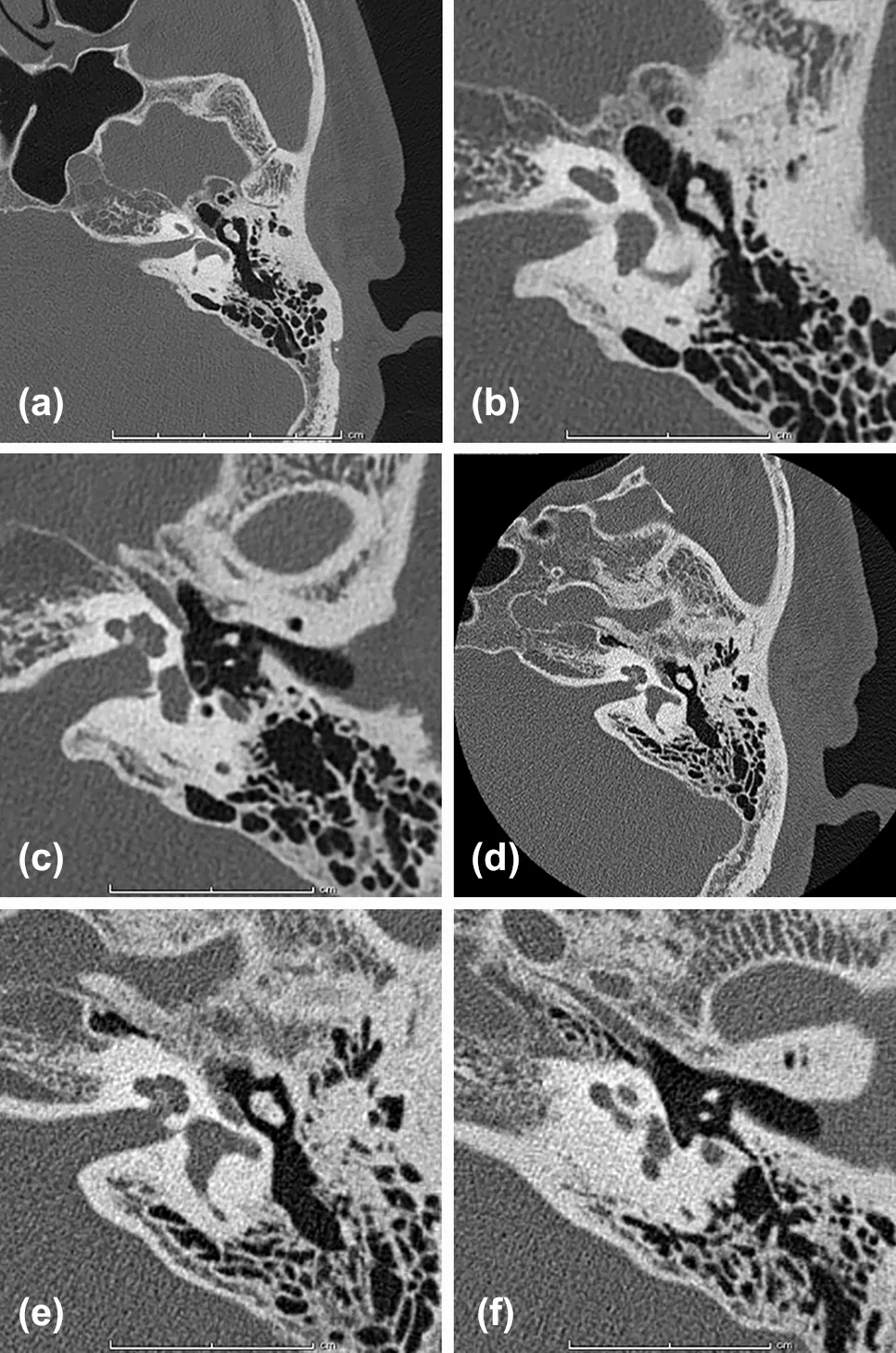
PCD-CT axial images of the left temporal bone CT at standard dose (**a**–**c**). The visual scores for both the incudomalleolar joint (**b**) and stapes (**c**) by two reviewers were 4. EID-CT axial images of the left temporal bone CT at standard dose (**d**–**f**). The visual scores for the incudomalleolar joint (**e**) and stapes (**f**) by two reviewers were both 2 PCD-CT, photon-counting detector computed tomography; EID-CT, energy-integrating detector computed tomography

The PCD-CT images and EID-CT images, both at standard dose, are shown in Fig. 3. The mean visual scores of PCD-CT images were significantly higher than EID-CT in all four regions (3.73 vs. 2.56 for the incudomalleolar joint, 3.75 vs. 2.63 for the stapes, 3.54 vs. 2.52 for the cochlea, and 3.58 vs. 2.46 for the facial nerve canal; all *P* values < 0.001) (Table 3). The agreement in assessment scores between readers was moderate, with the highest value (κ = 0.55) observed in the incudomalleolar joint.

**Table 3:** Mean visual scores of both PCD-CT and EID-CT at standard dose during clinical evaluation

	PCD-CT at standard dose	EID-CT at standard dose	Mean difference (95% CI)	P value	Weighted κ
Incudomalleolar joint	3.73 ± 0.25	2.56 ± 0.58	1.17 (0.90, 1.43)	< 0.001	0.55
Stapes	3.75 ± 0.33	2.63 ± 0.49	1.13 (0.88, 1.37)	< 0.001	0.38
Cochlea	3.54 ± 0.36	2.52 ± 0.43	1.02 (0.79, 1.25)	< 0.001	0.21
Facial nerve canal	3.58 ± 0.32	2.46 ± 0.41	1.13 (0.91, 1.34)	< 0.001	0.31

PCD-CT, photon-counting detector computed tomography; EID-CT, energy-integrating detector computed tomography; CI, confidence interval

### Phantom experiment to determine dose reduction limit with PCD-CT

**Fig. 4:**
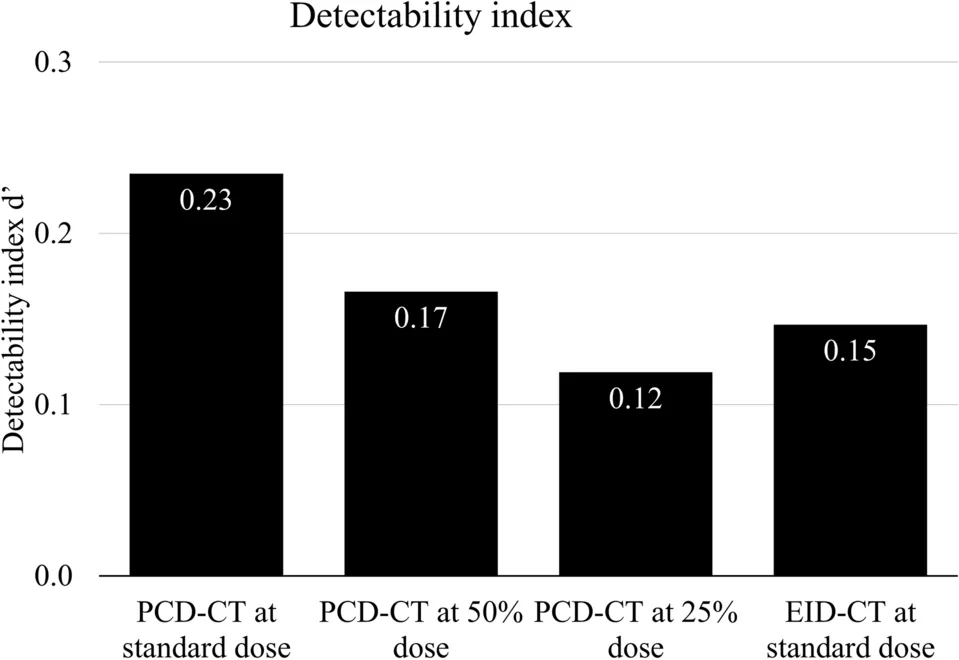
Detectability index of the representative images acquired with PCD-CT at standard dose, PCD-CT at 50% of standard dose, PCD-CT at 25% of standard dose, and EID-CT at standard dose PCD-CT, photon-counting detector computed tomography; EID-CT, energy-integrating detector computed tomography

The d’ values for each imaging protocol are shown in Fig. 4. While the PCD-CT images at 25% dose resulted in a lower d’ value compared to EID-CT at standard dose (0.12 vs. 0.15), the PCD-CT images at 50% dose showed a higher value (0.17). Therefore, the dose reduction used in the clinical evaluation was determined to be 50%.

### Clinical evaluation of PCD-CT images at the determined reduced dose

**Fig. 5:**
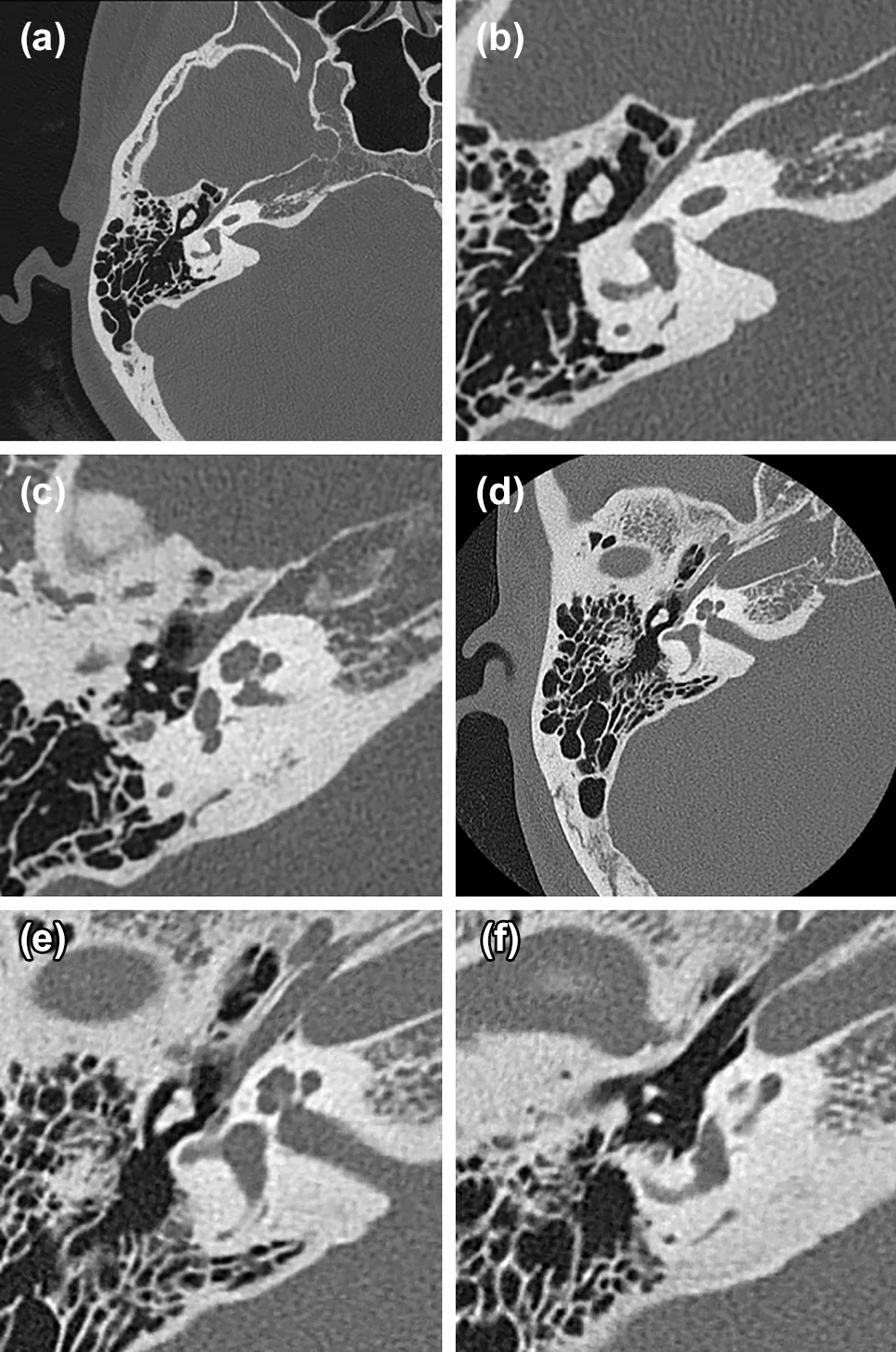
PCD-CT axial images of the right temporal bone CT at 50% doses (**a**–**c**). The visual scores for the incudomalleolar joint (**b**) and stapes (**c**) by two reviewers were both 4. EID-CT axial images of the right temporal bone CT at standard dose (**d**–**f**). The visual scores for the incudomalleolar joint (**e**) and stapes (**f**) by two reviewers were 2 and 3, respectively PCD-CT, photon-counting detector computed tomography; EID-CT, energy-integrating detector computed tomography

The images with PCD-CT at 50% dose and EID-CT at standard dose are shown in Fig. 5. The mean visual scores of PCD-CT images at 50% dose and EID-CT images at standard dose, and the 95% CI of the differences between the two images are shown in Table 4. The mean visual scores of PCD-CT images at 50% dose were equivalent in all four regions to EID-CT images at standard dose (3.58 vs. 3.12 for the incudomalleolar joint, 3.46 vs. 3.19 for the stapes, 3.50 vs. 3.08 for the cochlea, 3.58 vs. 3.27 for the facial nerve canal). The agreement in assessment scores between readers was moderate, with the highest value (κ = 0.42) observed in the incudomalleolar joint.

**Table 4:** Mean visual scores of PCD-CT at 50% of the standard dose and EID-CT at standard dose during clinical evaluation

	PCD-CT at 50% dose	EID-CT at standard dose	Mean difference (95% CI)	Weighted κ
Incudomalleolar joint	3.58 ± 0.40	3.12 ± 0.36	0.46 (0.15, 0.77)	0.42
Stapes	3.46 ± 0.48	3.19 ± 0.48	0.27 (-0.12, 0.66)	0.37
Cochlea	3.50 ± 0.35	3.08 ± 0.40	0.42 (0.12, 0.73)	0.25
Facial nerve canal	3.58 ± 0.40	3.27 ± 0.48	0.31 (-0.05, 0.67)	0.17

PCD-CT, photon-counting detector computed tomography; EID-CT, energy-integrating detector computed tomography; CI, confidence interval

## Discussion

The findings of this study demonstrated that the image quality of PCD-CT at standard dose was superior to that of EID-CT at standard dose in all four evaluated regions. Furthermore, the phantom study indicated a 50% reduction of the standard dose without diminishing imaging quality with PCD-CT. This finding was subsequently validated in the clinical study and constitutes the principal novelty of this study.

High-resolution images with PCD-CT have been shown in the field of cranial and spinal CT angiography [5], bone imaging [8], chest imaging [13], abdominal imaging [14], and coronary imaging [15, 16]. For example, Inoue et al. found that PCD-CT provided high-resolution chest images, giving the reader greater confidence in finding reticular shadows, ground-glass opacities, and mosaic patterns in patients with suspected interstitial pneumonia [13]. Mannil et al. reported that PCD-CT showed superior delineation of the coronary stent lumen using identical imaging protocols and reconstruction conditions to EID-CT [15].

In addition to high-resolution imaging, reduced radiation dose is another benefit of PCD-CT. It has been reported that the risk of brain tumors and leukemia increases when pediatric patients are exposed to radiation exceeding a certain amount [17]. In this context, the higher medical radiation exposure in Japan than in other countries [18] is an important concern, especially for pediatric patients. Baffour et al. reported that PCD-CT provides excellent delineation of bony structures in the shoulder and pelvis at a 31–47% lower radiation dose compared to EID-CT [7]. Donuru et al. reported that PCD-CT can significantly reduce the radiation dose while maintaining image quality and noise in plain chest CT compared to EID-CT [19]. In the abdomen, PCD-CT at low dose showed reduced noise, higher signal-to-noise ratio, better subjective image quality, and clearer abdominal microstructures, such as the ureters and mesenteric vessels [20].

For the temporal region, a comparison of EID-CT and PCD-CT of cadaveric temporal bones showed that PCD-CT had significantly lower image noise and was superior in depicting the fine anatomy [21]. Additionally, in clinical cases, temporal bone CT images with PCD-CT provide better visualization of bone anatomical structures, such as the tympanic membrane and cochlear meatus [9, 22]. Furthermore, PCD-CT provides detailed three-dimensional images that may improve diagnostic accuracy [23]. In a retrospective study of pediatric temporal bone CT, PCD-CT had a higher image quality rating at a lower dose than EID-CT [24]. In the diagnosis of superior semicircular canal dehiscence (SSCD), EID-CT, compared with PCD-CT, tended to overestimate the presence of SSCD and underestimate the thickness of the bone overlying the SSC [25]. Dose reduction strategies must be evaluated carefully to ensure that diagnostic performance is not compromised. For preoperative assessment, in particular, accurate and complete visualization of the stapes and the oval window is of critical clinical importance, as these findings directly influence surgical decision-making. Conversely, in situations where repeated imaging is anticipated—such as postoperative follow-up or monitoring of chronic otologic diseases—dose reduction while preserving sufficient diagnostic information may be particularly meaningful in clinical practice.

Akazawa et al. demonstrated that UHR-CT with step-and-shoot acquisition and limited z-axis coverage enables precise measurement of the stapes footplate in otosclerosis [3]. Their protocol was optimized to achieve maximal spatial resolution within a restricted anatomical region. Although the use of step-and-shoot acquisition may potentially improve image quality with EID-CT, its application to temporal bone imaging is unavailable with the PCD-CT used in this study. Therefore, to maintain methodological consistency, helical acquisition was selected for both of the two CT systems evaluated in the present study. In addition, step-and-shoot acquisition may not be sufficient for comprehensive evaluation of the entire temporal bone, including the mastoid air cells, since it typically limits the scan range to approximately 4 cm. This consideration was incorporated in the study design.

This study had several limitations. The clinical evaluation was conducted with a small population at a single center. Visual assessments used in clinical cases were subjective and performed by only two observers,resulting in limited inter-observer agreement, as reflected by the κ values. Only limited levels of dose reduction were assessed in the phantom study. In this study, a 1024 matrix was used for magnified reconstruction images. Considering the effective spatial resolution of the scanners, this setting may have been excessive. Furthermore, from a practical perspective, and considering the potential burden on PACS and other medical resources, a 512 matrix setting may be sufficient for routine clinical practice. The potential effects of filter implementation were not investigated, although a previous phantom and cadaver study showed that PCD-CT using a tin filter in the temporal bone reduced the dose by 83% compared with EID-CT [26]. Whether the application of high atomic number filters, such as a tin filter, could further reduce radiation dose needs to be investigated in future studies.

In conclusion, temporal bone images with PCD-CT preserve image quality even when the dose is reduced by 50% of the standard dose.
